# Prevalence of Genetic Variants and Deep Phenotyping in Patients with Thoracic Aortic Aneurysm and Dissection: A Cross-Sectional Single-Centre Cohort Study

**DOI:** 10.3390/jcm13020461

**Published:** 2024-01-14

**Authors:** Adrian Mahlmann, Nesma Elzanaty, Mai Saleh, Marc Irqsusi, Ardawan Rastan, Jennifer Lynne Leip, Christian-Alexander Behrendt, Tamer Ghazy

**Affiliations:** 1 Department of Internal Medicine III, University Hospital Carl Gustav Carus, Technische Universität Dresden, 01307 Dresden, Germany; mahlmanna@kkh-hagen.de; 2 Centre for Vascular Medicine, Clinic of Angiology, St.-Josefs-Hospital, Katholische Krankenhaus Hagen gem. GmbH, 58097 Hagen, Germany; 3 Department of Medical Physiology, Tanta Faculty of Medicine, Tanta University, Tanta 31527, Egypt; nesma.elzanaty@med.tanta.edu.eg; 4 Department of Chest Diseases, Tanta Faculty of Medicine, Tanta University, Tanta 31527, Egypt; mai.saleh@med.tanta.edu.eg; 5 Department of Cardiac Surgery, Marburg University Hospital, Philipps University of Marburg, 35043 Marburg, Germany; marc.irqsusi@med.uni-marburg.de (M.I.); rastan@uni-marburg.de (A.R.); 6 Northeastern University, Boston, MA 02115, USA; 7 Department of Vascular and Endovascular Surgery, Asklepios Clinic Wandsbek, Asklepios Medical School, 20099 Hamburg, Germany; behrendt@hamburg.de; 8 Brandenburg Medical School Theodor Fontane, 16816 Neuruppin, Germany

**Keywords:** thoracic aortic disease, phenotype, genetic mutation, genetic variant, aneurysm, dissection

## Abstract

Background: There is a paucity of evidence on people with thoracic aortic aneurysm and dissection. We aimed to determine the prevalence of genetic variants and their associations with phenotypes. Methods: In this cross-sectional single-centre cohort study of consecutive patients who underwent endovascular or open-surgical repair of thoracic aortic aneurysm and dissection, genetic analysis was performed using four-stage Next Generation Sequencing, and findings were confirmed with Sanger sequencing. We collected personal and family history on comorbidities, clinical examination, anthropometrics, skeletal deformities, joint function, and ophthalmological measures. Cardiovascular risk and phenotype scores were calculated. Results: Ninety-five patients were eligible (mean age 54 ± 9 years, 70% males, 56% aortic dissection). One-fifth had a family history of aortic disease. Furthermore, 95% and 54% had a phenotype score of ≤5 and ≤2, respectively. There were no significant differences in the distribution of phenotype characteristics according to age, sex, aortic pathology, or performed invasive procedures. Genetic variants of uncertain significance were detected in 40% of patients, with classic mutations comprising 18% of all variants. We observed no significant association with cardiovascular and phenotype scores but with higher joint function scores (*p* = 0.015). Conclusion: Genetic variants are highly present in clinically relevant aortic pathologies. Variants appear to play a larger role than previously described. The different variants do not correlate with specific phenotypes, age, pathology, sex, or family history.

## 1. Introduction

Thoracic aortic aneurysm and dissection (TAAD) is a relatively low-frequency disease affecting 5–10 per 100,000 of the global population [1]. Aneurysms stay asymptomatic in up to 95% of cases until a life-threatening dissection or rupture of the aorta occurs [1,2].

Early detection of TAAD and precise classification of the underlying aetiology may help to optimise lifelong follow-up and patient-tailored therapy plans [3]. Whether it is cardiovascular atherosclerosis, connective tissue disease, trauma, or inflammatory vascular wall changes, it has an impact on many decision pathways [4,5].

Generally, TAADs can be syndromic (e.g., Marfan syndrome (MFS), Loeys-Dietz syndrome (LDS), or Ehlers-Danlos syndrome (EDS)) or non-syndromic. Despite having different clinical pictures and prognoses, both syndromic and non-syndromic forms are often based on genetic variants [2].

Since the establishment of high-throughput sequencing, also known as Next Generation Sequencing (NGS), knowledge of pathogenic variants has been accumulated [6]. Yet, the prevalence of genetic variants in patients with clinically relevant aortic pathology indicating aortic therapy has not been fully studied. Furthermore, in the presence of modern NGS, the current correlation of genetic variants and the presence of phenotypical features typical for syndromic TAAD need to be re-evaluated.

The aim of the present study was to determine the prevalence of genetic variants in patients with TAAD who underwent invasive aortic repair. Furthermore, phenotype–genotype correlation was studied to reveal the prevalence of phenotypical characteristics in patients with confirmed genetic variants. This may help to understand its role in the justification of indications for human genetic testing using NGS.

## 2. Material and Methods

### 2.1. Ethical Statement

The study was reviewed and approved by the ethical committee board of the Technische Universität Dresden (decision number EK317082014). The indication for human genetic analysis was made by the Department of Angiology at the University Centre for Vascular Medicine. It was also confirmed by the cooperating practice for human genetics. STrengthening the Reporting of OBservational studies in Epidemiology (STROBE), the statement for the reporting of observational studies, has also been complied with [7].

### 2.2. Study Design and Cohort

In this cross-sectional single-centre cohort study, patients who were admitted to a tertiary reference centre in a university setting from 1 January 2008 to 30 June 2019 were screened for inclusion in the study. The inclusion criteria were age 18 or older, undergoing endovascular or open-surgical treatment for an index aortic pathology, and patient-informed consent. The exclusion criteria were aortic pathology due to inflammatory or traumatic genesis and patients over the age of 65 with no offspring.

### 2.3. Recruitment

A database screening for patients with aortic pathology who underwent an open-surgical or endovascular treatment from January 2008 through June 2019, according to the documented diagnosis code (World Health Organisation International Classification of Diseases, WHO-ICD), was carried out. The list of suitable ICD and procedural codes is provided in the Supplementary Files. Eligible patients were subsequently contacted and asked for inclusion in this study. As part of the comprehensive sensitivity analyses and to determine the risk of selection bias, we used the anonymised data of excluded patients to compare the demographic data, risk factors, aortic pathology, performed procedures, and medications with the study cohort.

The included patients underwent personal and family history taking, clinical examination, including thorough phenotype analysis, and subsequent human genetic analysis as follows.

### 2.4. Collection of History Data, Clinical Parameters, and Risk Score Calculation

Demographic data were collected from patients’ files and directly from patients. For age-group analysis, age was divided into three groups: patients under 45 years (group 1), between 45 and 60 years (group 2), and over 60 years (group 3).

Further history and clinical data with possible associations with connective tissue diseases were collected during a comprehensive clinical examination and anamnesis of medical history.

#### 2.4.1. Family History

Positive history was defined as confirmed connective tissue disease or clinically suspected due to confirmed aortic disease in relatives of the 1st, 2nd, or 3rd degree.

#### 2.4.2. Data Collection/Categories for Risk Score Calculation

##### Stature and Extremities:

Tall stature (defined as >97th percentile of the normal range based on normal population data, where the normal range is defined according to age and population group between the 3rd and 97th percentile); arm span/body length ratio >1.05, arachnodactyly, striae, and increased skin elasticity.

##### Skeletal Deformities:

Thoracic deformities (pectus carinatum/excavatum), scoliosis, foot deformities (pes planus and pes equinovarus), and protrusio acetabuli.

##### Craniofacial Abnormalities:

Cleft palate, high palate, uvula bifida, narrow lips, dolichocephaly, craniosynostosis, midface hypoplasia, micrognathia, and hypertelorism.

##### Joint Function:

Positive Murdoch or Steinberg signs, Beighton score (hypermobility), and joint dislocations.

##### Ophthalmologic Pathologies:

Lens luxation/ectopia, myopia >3 dioptres, glaucoma, cataracts, retinal detachment, enophthalmos, iris hypoplasia, and blue sclera.

##### General Diagnoses:

Hernia, spontaneously developed pneumothorax, and dural ectasia.

##### Cardiovascular Pathology and Cumulative Risk Score:

Bicuspid aortic valve and atrial septal defect (ASD).

These seven defined phenotype categories with representative clinical features/risk characteristics were used for scoring. In each category, one point was awarded for each risk characteristic and summed up for total points for this category. In the category of joint function, we also used established systemic scoring scales such as Beighton (max. of nine points possible) and the Murdoch or Steinbach sign (each sign is worth one point). If more than two signs were fulfilled in the Beighton score, only this scale was regarded as the total point score in the category of joint function. The higher the cumulative score, the higher the clinical probability of the possible presence of a connective tissue disease.

### 2.5. Further Comorbidities

The following comorbidities were recorded: arterial hypertension, defined as systolic blood pressure values of 130 mmHg or more, and/or diastolic blood pressure of 90 mmHg or more, and/or indicating medical treatment; clinically relevant hyperlipoproteinemia, defined as hyperlipoproteinemia indicating oral medical therapy; Diabetes mellitus indicating medical treatment (either oral antidiabetic or Insulin therapy); renal impairment, defined as a glomerular filtration rate (GFR) <60 mL/min; active or past history of smoking; coronary heart disease (CHD); lower extremity peripheral arterial disease (PAD); documented carotid stenosis; history of stroke; chronic obstructive pulmonary disease (COPD) indicating medical treatment.

### 2.6. Molecular Genetic Analysis

The molecular genetic analysis was obtained from ethylenediaminetetraacetic acid (EDTA) blood using the Next Generation Sequencing (NGS) panel analysis method. After capture-based enrichment of the genetic material, the NGS four-stage sequencing method (IMiSeq Desktop Sequencer, llumina Inc., San Diego, CA, USA) was applied. If a gene change was detected, it was confirmed using conventional Sanger sequencing. In addition to Multiplex Ligation-dependent Probe Amplification, the Sanger method was also used if a follow-up analysis became necessary due to ambiguous NGS results.

Due to the genetic heterogenicity of the connective tissue disease spectrum, nine genes were initially panel-examined and evaluated, according to the specification of the German health authorities for examining genes associated with connective tissue diseases with possible involvement of the thoracic aorta. The examined gene loci were: *ACTA2*, *COL3A1*, *FBN1*, *MYH11*, *MYLK*, *SMAD3*, *TGFB2*, *TGFBR1,* and *TGFBR2*. Due to the design of clinical routine diagnostics and the defined criteria for examination of further gene loci by the health insurance system, such as in cases of the presence of phenotypical features or positive family history, the genetic diagnostics were expanded after patient consent was provided to include the analysis of the following genes: *AEBP1*, *BGN*, *COL1A1*, *COL4A5*, *COL5A1*, *COL5A2*, *EF-EM P2*, *ELN*, *FBLN5*, *FBN2*, *FLNA*, *FOXE3*, *GATA5*, *LOX*, *MAT2A*, *M FAP5*, *NOTCH1*, *NOTCH3*, *PLOD1*, *PRKG1*, *RPL26*, *SKI*, *SLC2A10*, *SMAD4*, *SMAD6*, *TAB2*, and *TGFB3*. The focus of the variants was fixed on the defined genes of interest listed. Thus, whole genome sequencing was not performed.

### 2.7. Data Acquisition and Cooperation Partners

General patient data, as well as diagnoses and findings, were recorded using the electronic hospital information system used at the Dresden University Hospital in Dresden, Germany. These included performed interventions, documentation of the clinical and image morphological data, structured follow-up, and the multidisciplinary vascular conferences of the disciplines of angiology, vascular surgery, interventional radiology, and cardiac surgery at the Dresden Heart Centre, which were in accordance with established standards at the University Vascular Centre of the Medical Clinic and Policlinic III of the Carl Gustav Carus University Hospital.

Cardiac surgical intervention data and the corresponding documentation were acquired from the Department of Cardiac Surgery at the Dresden Heart Centre. Human genetic clinical examination, as well as routine blood tests and subsequent human genetic analysis, were performed by outpatient specialists in human genetics from the group practice for human genetics (Gutenberg, Str. 5, 01307 Dresden, Germany; existing cooperation agreement with the University Hospital Carl Gustav Carus).

### 2.8. Statistical Analysis

Descriptive analyses were provided using absolute or relative frequencies. The mean and standard deviations were used to present continuous variables. Frequency differences were analysed using chi-square text for nominal scale variables and the Kruskal–Wallis or Mann–Whitney U test for ordinal scale variables. To assess the correlation between age and cardiovascular risk factors, the Kendall’s Tau correlation coefficient was calculated. Continuous variables were compared using the *t*-test. Results were considered statistically significant when *p* < 0.05. If the probability of error, *p*, yielded values between 0.05 and 0.1 (0.05 ≤ *p* < 0.1), the results were considered to be showing a trend. IBM SPSS software (IBM SPSS Statistics Version 28, IBM, Armonk, NY, USA) was used for the statistical analysis.

## 3. Results

### 3.1. Patient Cohort, Demographic Data, and Representability of the Study Cohort

A total of 1334 patients with aortic disease of any aetiology were initially screened for eligibility. Therefore, 716 patients were eligible according to the inclusion and exclusion criteria. Ultimately, 116 patients gave their full consent, while 95 patients provided complete consent and were included in the current study.

The comparative analysis between the study cohort and the patients who met the inclusion criteria but were excluded due to failure of patient consent or an incomplete data set showed no significant differences between both cohorts regarding age, sex, performed procedures, arterial hypertension, history of smoking, hyperlipoproteinemia, diabetes mellitus, coronary heart disease (CHD), carotid stenosis, and peripheral artery disease (PAD) (Supplementary Table S1). The analysis showed that the study cohort had more aortic dissections than the fully screened cohort with fulfilled inclusion/exclusion criteria (57.9% vs. 44.6%, *p* = 0.009). Both cohorts showed no significant differences in medical therapy with angiotensin-converting enzyme inhibitors, calcium antagonists, beta blockers, antiplatelet drugs, and other antihypertensives. More study cohort patients were on oral anticoagulants than the excluded cohort (36.8% vs. 29.7%, *p* = 0.021).

Data analysis of the study cohort showed a mean age of 54 ± 9 years with a 70.5% male quota. In the age group of 45 to 65 years, aortic dissection was represented more frequently than aortic aneurysm (Figure S1, Supplementary Materials). Arterial hypertension was documented in 83.2% of patients, a history of smoking in 33.7%, and hypercholesterolemia in 32.6%. A total of 57.9% of patients suffered aortic dissections, and 42.1% had aortic aneurysms. Further distribution analysis of pathology according to age groups is depicted in Supplementary Figure S1. A total of 68% of patients underwent open aortic surgery, and 31.6% were treated endovascularly.

### 3.2. Family History Analysis and Distribution of Phenotypical Characteristics

A positive family history was documented in 20% of patients (more common in women than in men; *p* = 0.056). A positive history in first-degree relatives was confirmed in 4.2% of aneurysm patients and 2.1% of dissection patients. There was a higher prevalence of genetic variants in patients with a positive family history (*p* = 0.028).

**Figure 1:**
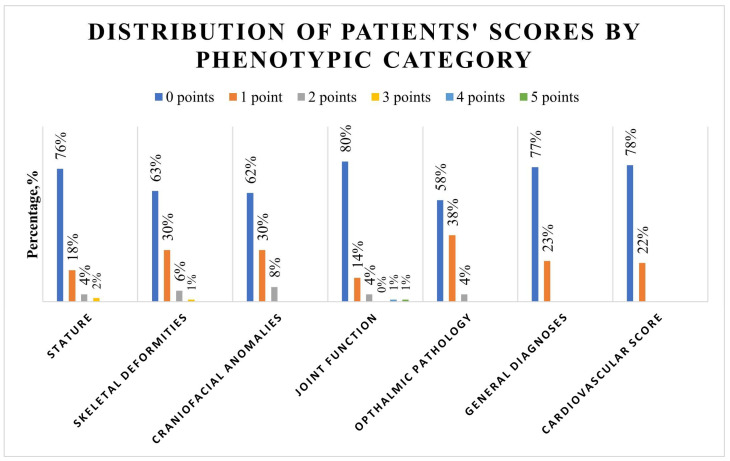
Distribution of patients’ phenotype scores: For each category, a total value from all risk points was created for every patient. One point was awarded for each risk characteristic, and the total risk score was calculated. In the joint function category, the higher point value from either the Murdoch or Steinberg signs with one point each (maximum two) or the total value of the Beighton score (maximum nine points) was used.

A total of 54% of patients showed a total phenotypic score of two or less, and 92.6% showed a score of five or less. The detailed score distribution across the study population is depicted in Supplementary Figure S2. The phenotypical category analysis (Figure 1, Supplementary Table S2) showed that tall stature was present in 6.3% of patients.

The arm-span-to-body length ratio was above normal in 14.7% of patients. In skeletal deformities, pes planus was the most present feature, with a prevalence of 20.0%, followed by scoliosis in 13.7% of patients. Thin lips were the most prevalent craniofacial anomaly and were present in 25.3% of patients, followed by a high palate prevalence of 14.7%. All joint function signs were present in less than 10%, with the highest prevalence of 9.5% documented for the Murdoch sign. Myopia was present in 14.7% of patients, and enophthalmos in 11.6%. A total of 23% of patients showed a positive history of hernia, and 17.9% had a bicuspid aortic valve. Further subgroup analysis showed no significant difference in the distribution of phenotypical characteristics according to age group, sex, aortic pathology, or performed procedure (Table 1).

### 3.3. Analysis of Genetic Variants and Correlation with Phenotype

**Figure 2:**
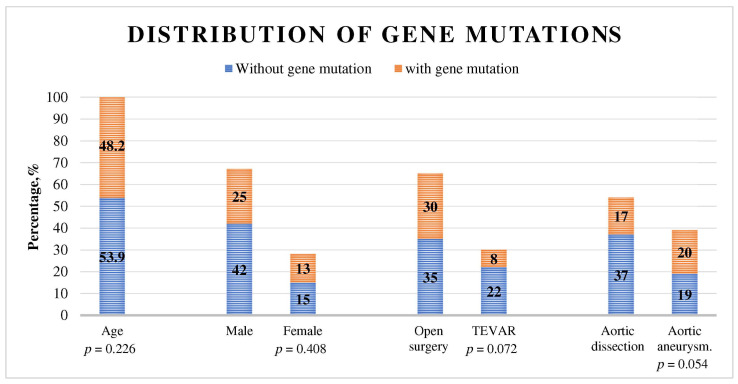
Distribution of gene mutations across demographic and clinical patients’ subgroups.

The genetic analysis confirmed gene variants in 40% of all patients. Classic mutations comprised 18.4% of all variants, while the other variants of uncertain significance (VUS) constituted 81.6% of detected variants. The distribution of genetic variants did not differ significantly across age or sex. The analysis showed a trend towards a higher prevalence of genetic variants in aortic dissections and in patients undergoing open surgery (*p* = 0.054 and *p* = 0.072, respectively, Figure 2 and Table 2).

Genetic variants were found in thirteen genes. *FBN1* gene variants comprised 32.5% of total variants, 7.5% of which were classic variants and 25% were other variants. *MYH11* and *TGFB2* followed and comprised 20% and 10% of total variants, respectively. Other gene variant percentages were under 10% each (Table 2). Simultaneous variants in two genes were detected in three patients (*FGFB2* and *MYH11* genes; *MYH11* and *NOTCH1* genes; and *FBN1* and *SMAD3* genes).

**Figure 3:**
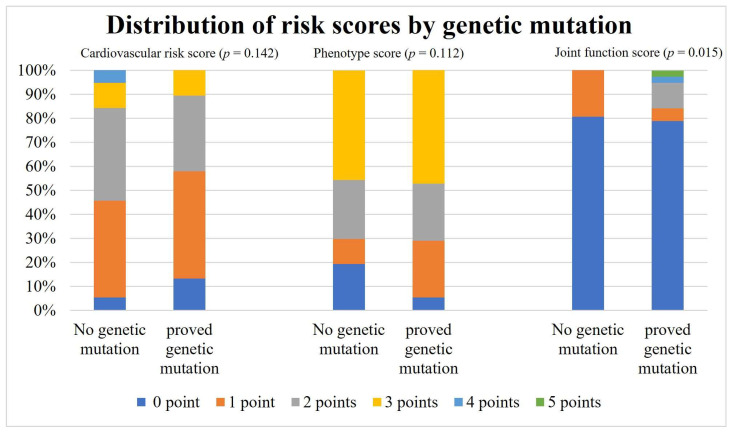
Distribution of risk scores by genetic mutation.

The distribution of different risk scores by genetic variant is depicted in Figure 3.

For each risk score, a total value from all risk points was created for each patient. One point was awarded for each risk characteristic, and the total risk score was calculated. The percentage of each patient group with the same score is depicted with a different colour in each column.

Cardiovascular scores and phenotype scores did not show a significant difference in distribution between patients with or without genetic variants (*p* = 0.140 and *p* = 0.110, respectively). A significant difference was found in joint function score analysis, with a higher joint function score in patients with genetic variants (*p* = 0.015). The detailed results of the analysis are listed in Table 3.

### 3.4. Genetic Variants and Their Association with Genetic Disease

Two patients with *FBN1* gene variants showed the characteristics of Marfan syndrome. Both patients carried heterozygous variants. Two patients showed a high probability of Loeys-Dietz syndrome class 4; one of them carried a heterozygous *TGFBR1* gene variant, and the other carried a heterozygous deletion of the *TGFB2* gene. An Ehlers-Danlos syndrome of the vascular type was documented in one patient with a missense variant in the *COL2A1* gene.

Furthermore, one patient with a heterozygous duplication of the *MYH11* gene and a heterozygous variant in the *NOTCH1* gene showed the microduplication syndrome (16p13.11); a patient with the heterozygous variant of the *NOTCH3* gene showed CADASIL syndrome; a non-syndromic craniosynostosis was documented in a patient with the heterozygous variant of the NOTCH1 gene; and a patient with karyotype 45 X0 suffered from Turner syndrome.

All other detected gene variants were unclassified variants, variants with low clinical relevance, or variants without known clinical relevance (classes 1 to 3 according to Plon et al. [8]).

## 4. Discussion

In this study, human genetic analysis was abnormal in 40% of all cases, with 87.5% of all identified variants assigned to category A genes, which represent a relevant risk for thoracic aortic aneurysm and dissection (TAAD) according to the classification published by Renard et al. [9]. Multiple syndromes were detected, including Marfan syndrome, Loeys-Dietz syndrome, and Ehlers-Danlos syndrome of the vascular type. It is significant to note that most of the variants were of unclear significance. However, this is compared to studies with designs that were similar to the presented study with their inclusion of non-selected TAAD patients [10,11]. Studies that showed a higher prevalence of pathologic variants were those with designs that only considered TAAD patients under certain inclusion criteria [12,13,14,15,16,17,18,19]. The high prevalence of variants of unclear significance in non-selected TAAD patients denotes that variants of unclear significance might play a larger role than is currently known. This should be thoroughly examined in future studies.

An important finding of this study was that there was no significant correlation between phenotypic and genetic variants. Only the joint movement score was significantly correlated with genetic variants. Further analysis confirmed that the genetic variant distribution was independent of age, sex, pathology, or cardiovascular risk. This confirms the importance of genetic testing, irrespective of phenotype, demographic data, or cardiovascular risk. To the best of our knowledge, this is the first report systemically investigating the phenotypical features of all-comer TAAD patients and their correlation to the genotype. In the study by Pope et al., only people with suspected hereditary TAAD, not those with sporadic TAAD, were included in the study. Clinical data were collected, but not systematically. A clinical study of their participants was carried out by Duan et al. To rule out hereditary connective tissue disease, this study specifically included people with marfanoid characteristics or lens ectopy, not just those with TAAD, of which only 68.2% were affected. This could explain higher proportions of striking clinical features [19]. A correlation to the genotype was not recorded. However, the authors found a significant association between striae distensae and TAADs. The presence of striae distensae could therefore be a clinical clue to look for TAAD in individuals at risk [19]. In the study cohort of Wooderchak-Donahue et al., the characteristics of lens ectopia and some musculoskeletal findings (dural ectasia, reduced elbow extension, and marfanoid habitus) were more conspicuous in people with the genetic variant. In contrast, skin changes and musculoskeletal features such as hypermobile joints, enlarged limbs, pes planus, and hindfoot deformities were more common in the negative cohort [14]. In the study by Campens et al., the presence of syndromic features significantly increased (up to threefold) the likelihood of genetic variant detection [15]. As a conclusion, we believe that although the presence of typical phenotypical features increases the probability of genetic variants, their absence should not be an exclusion criterion in the decision-making algorithm for indicating genetic testing.

Although familial studies have shown a tenfold increased incidence rate in first-degree relatives with a family history of thoracic aortic aneurysm [20], other studies have reported that sporadic TAADs without any evidence of a hereditary association could be based on genetic mechanisms. Therefore, gene analysis could be indicated in these patients [2]. In a study by Guo et al., 28% of subjects with sporadic thoracic aortic dissection ages ≤56 years presented at least one variant of unclear significance, and 9.3% carried a genetic variant in any of the eleven syndromic or familial TAAD genes, significantly more than the control [21]. In the study published by Renner et al., the diagnostic yield was not significantly higher in people with a positive family history than those without [18]. This concurs with the results of our study, which showed a comparable genetic variant prevalence with no correlation with family history. An explanation for this might be that most of the detected genetic variants were of unclear significance. It is important to note that although over 37 genes are known to be associated with hereditary TAADs [22], only around 30% of familial non-syndromic TAAD cases have a genetic variant in these genes. This suggests that most of the genetic basis of these thoracic aortic aneurysms and dissections remains undiscovered [22,23]. Further genetic testing of family members to examine the prevalence of detected genetic variants and their clinical relevance is crucially needed.

## 5. Limitations

In addition to many strengths, there were also limitations to this study. First, the study was limited to a single centre and was cross-sectional by design. The totality of the data in such epidemiological studies on genetic variants depends on voluntary participation. Due to the high value of biogenetic data and informational self-determination in the global discussion about individual privacy, it is not taken for granted that people will always consent. This challenge has appeared in numerous cohort studies in the past. Although we conducted comprehensive sensitivity analyses and are confident that excluded patients were not systematically different from the study cohort, selection bias and residual confounding cannot be omitted. Regarding the different timespan of TAAD onset and performing the genetic examination of the recruited patients, the spontaneous development of new mutations due to surgical interventions, environmental changes, and ageing has to be acknowledged. Mutations detected long after the onset of TAAD might result in differences in the distribution of mutations compared to patients with earlier genetic examinations for a nearby time point of primary TAAD onset. In addition, further testing for supplemental genes was only performed on a subset of patients with phenotypical features or a positive family history. This may produce a tendency bias in patient selection and distort the results, as the other patients may also have mutation possibilities in these genes. The solicitation of family history came only from the participants themselves and did not include any additional information from their relatives. Self-reported medical information remains a challenge in epidemiological studies, but we designed the variables in a robust way to avoid another bias. Finally, the gene palette examined was limited. Future research should consider expanding the participant base to include more than one centre, expanding the gene palate, and involving participants’ relatives in the family history solicitation process.

## 6. Conclusions

Genetic variants are highly present in clinically relevant aortic pathology. Variants of unknown relevance seem to play a larger role than previously known. These genetic variants do not correlate with a specific phenotype, age group, pathology, sex, or family history. Therefore, extending genetic testing to all patients with clinically relevant aortic pathology should be considered, regardless of these factors.

## Figures and Tables

**Table 1:** Subgroup analysis and its correlation with phenotype.

Variable	No Phenotypical Signs	Present Phenotypical Signs	*p*-Value
	Number (percentage)	Number (percentage)	
Age			0.783
under 45 years	3 (11.5%)	23 (88.5%)	
45–65 years	8 (16.0%)	42 (84.0%)	
over 65 years	2 (10.5%)	17 (89.5%)	
Sex			0.712
males	9 (13.4%)	58 (86.6%)	
females	4 (14.3%)	24 (85.7%)	
Aortic pathology			0.413
aortic aneurysm	6 (15.0%)	34 (85.0%)	
aortic dissection	8 (14.5%)	47 (85.5%)	
Treatment			0.478
surgical	10 (15.4%)	55 (84.6%)	
endovascular	3 (10.0%)	27 (90.0%)	

**Table 2:** Distribution of gene mutations across the study cohort.

Name of the Gene	Number of Mutation Detections (*n*)	Proportion of the Total Number of Mutation Detections (in %)	Classic Mutation (*n*)	Percentage of Classic Mutations to Total Number of Evidence of Mutation (in %)	Mutation Variant (*n*)	Percentage of Mutation Variants in total Number of Mutation Detections (in %)
*FBN 1*	13	32.5	3	7.5	10	25
*COL3A1*	1	2.5	1	2.5	0	0
*SMAD3*	1	2.5	0	0	1	2.5
*TGFB2*	4	10.0	1	2.5	3	7.5
*TGFBR1*	2	5.0	0	0	2	5.0
*MYLK*	2	5.0	0	0	2	5.0
*MYH11*	8	20.0	0	0	8	20.0
*PRKG1*	1	2.5	0	0	1	2.5
*NOTCH* *3*	1	2.5	1	2.5	0	0
*NOTCH* *1*	3	7.5	0	0	3	7.5
*TGFBR2*	1	2.5	0	0	1	2.5
*ACTA2*	2	5.0	0	0	2	5.0
*SMAD6*	1	2.5	0	0	1	2.5

**Table 3:** Correlation of demographic data and risk factors with gene mutation.

Variable	Without Gene Mutation	With Gene Mutation	*p*-Value
	Mean (±standard deviation)	Mean (±standard deviation)	
Age (in years)	53.9 ± 10.5	48.2 ± 13.7	0.226
	Number (percentage)	Number (percentage)	
Males	42 (62.7%)	25 (37.3%)	0.408
Females	15 (53.6%)	13 (46.4%)	
Open surgery	35 (53.8%)	30 (46.2%)	0.072
TEVAR	22 (73.3%)	8 (26.7%)	
Aortic aneurysm	20 (50.0%)	20 (50.0%)	0.052
Aortic dissection	37 (67.3%)	18 (32.7%)	
Cardiovascular risk score			0.142
0 point	3 (5.3%)	5 (13.2%)	
1 point	23 (40.4%)	17 (44.7%)	
2 points	22 (38.6%)	12 (31.6%)	
3 points	6 (10.5%)	4 (10.5%)	
4 points	3 (5.3%)	0 (0%)	
Phenotype score category			0.122
Score = 0	11 (19.3%)	2 (5.3%)	
Score = 1	6 (10.5%)	9 (23.7%)	
Score = 2	14 (24.5%)	9 (23.7%)	
Score ≥ 3	26 (45.6%)	18 (47.4%)	
Joint function score			0.015
Score = 0	46 (80.7%)	30 (78.9%)	
Score = 1	11 (19.3%)	2 (5.3%)	
Score = 2	0 (0%)	4 (10.5%)	
Score = 3	0 (0%)	0 (0%)	
Score = 4	0 (0%)	1 (2.6%)	
Score = 5	0 (0%)	1 (2.6%)	

Abbreviations: TEVAR = Thoracic Endovascular Aortic Repair.

## Data Availability

Study data is unavailable due to privacy or ethical restrictions.

## Supplementary Materials

The following supporting information can be downloaded at: https://www.mdpi.com/article/10.3390/jcm13020461/s1.
