# Supplementary material for: Prevalence of Genetic Variants and Deep Phenotyping in Patients with Thoracic Aortic Aneurysm and Dissection: A Cross-Sectional Single-Centre Cohort Study
Source: J Clin Med. 2024 Jan 14;13(2):461. doi: 10.3390/jcm13020461 (PMC10816602; doi:10.3390/jcm13020461)
Supplement: Supplementary file 1 [file jcm-13-00461-s001.zip › jcm-2794544-supplementary.pdf]

Table S1

Demographic distribution and risk factors			
Parameter	Results<br>n = 95, study cohort	n = 716, totally screened with fulfilled inclusion / exclusion criteria	p-value
Age	54.9 ± 12.0	59.2 ± 8.2	0.832
Male sex	70.5%	68.7%	0.385
Aortic surgery	68.4%	72.1%	0.153
TEVAR	31.6%	27.9%	0.278
Main diagnosis TAD	57.9%	44.6 %	0.009
Main diagnosis TAA	42.1%	52.4%	0.007
Cardiovascular risk factors			
Arterial hypertension	83.2%	80.9%	0.532
(Ex-)nicotine	33.7%	35.1%	0.874
Hypercholesterolemia	32.6%	29.8%	0.756
Diabetes mellitus	8.4%	9.7%	0.544
CHD (coronary heart disease)	13.7%	12.3 %	0.223
Carotid stenosis or stroke	5.3%	6.1%	0.178
PAD (peripheral arterial occlusive disease)	4.2%	3.6%	0.159
Medications			
Beta blockers	94.7%	92.8%	0.656
ACE inhibitors/AT1 receptor antagonists	83.1%	81.7%	0.258
Platelet aggregation inhibitors	42.1%	40.9%	0.175
Calcium antagonists	41.1%	39.2%	0.288
Diuretics	40.0%	42.4%	0.731
Oral anticoagulation	36.8%	29.7%	0.021
Other antihypertensive drugs	21.1%	22.3%	0.410

Table S2

Distribution of phenotypical characteristics	
**Phenotypic characteristic**	**Result (in %)**
**Family History**	
Positive family history of connective tissue or aortic disease In first- to third-degree relatives	20%
1st degree relatives with aortic dissection	2.1%
1st degree relatives with aortic aneurysm	4.2%
**Stature**	
Normal height	91.6%
Normal Relationship from arm span to body length	85.3%
Ratio of arm span to body length above to the normal range	14.7%
Tall stature	6.3%
Striae	5.3%
Arachnodactyly	3.2%
Short stature	2.1%
Increased skin elasticity	1.1%
**Skeletal deformities**	
Pes planus	20.0%
Scoliosis	13.7%
Pectus carinatum	6.3%
Pectus excavatum	4.2%
Protrusion acetabuli	1.1%
Pes equinovarus	0%
**Craniofascial anomalies**	
Thin lips	25.3%
Higher palate	14.7%
Dolichocephaly	5.3%
Hypertelorism	1.1%
Cleft palate	0%
Craniosynostosis	0%
Midface hypoplasia	0%
Micrognathia	0%
Uvula bifida	0%
**Joint function**	
Murdoch sign	9.5%
Joint dislocations	5.3%
Steinberg sign	4.2%
Beighton Score 1	4.2%
Beighton Score 4	2.1%

Beighton Score 2	1.1%
**Ophthalmic pathologies**	
Myopia ≥ 3 diopters	14.7%
Enophthalmos	11.6%
Cataract	7.4%
Blue sclera	7.4%
Glaucoma	5.3%
Ablation retinae	0%
Iris hypoplasia	0%
Ectopia lentis	0%
**General diagnoses**	
Hernias	23.2%
Spontaneous pneumothorax	0%
Dura ectasia	0%
**Cardiovascular**	
Bicuspids aortic valve	17.9%
Atrial septal defect	4.2%

Figure S1

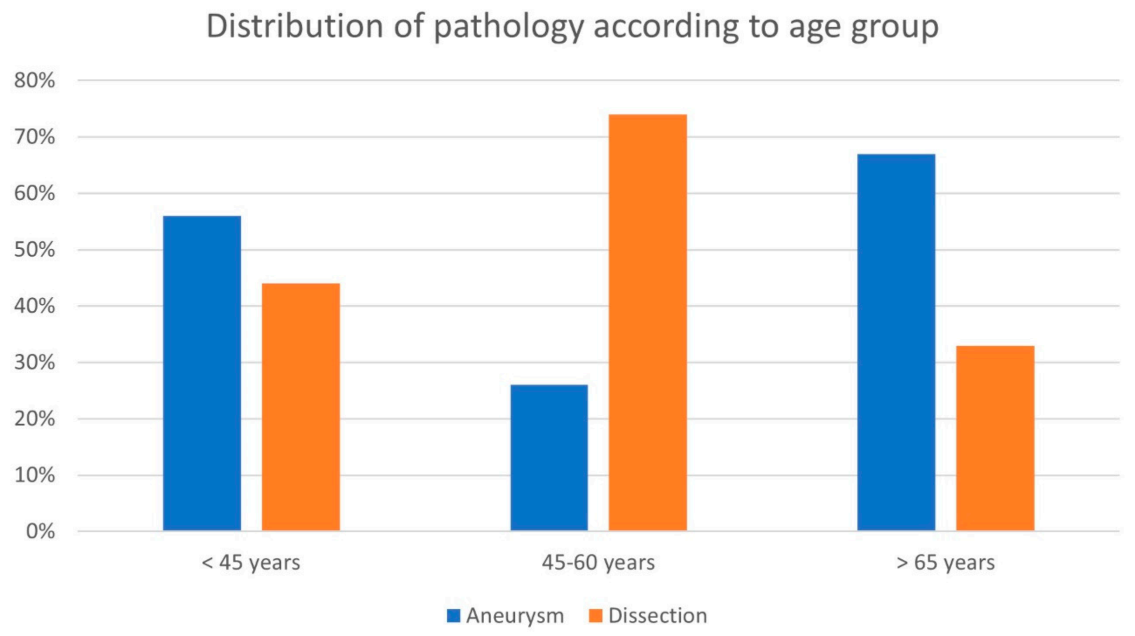
Distribution of pathology according to age group
80%
70%
60%
50%
40%
30%
20%
10%
0%
< 45 years
45-60 years
> 65 years
Aneurysm
Dissection

Figure S2

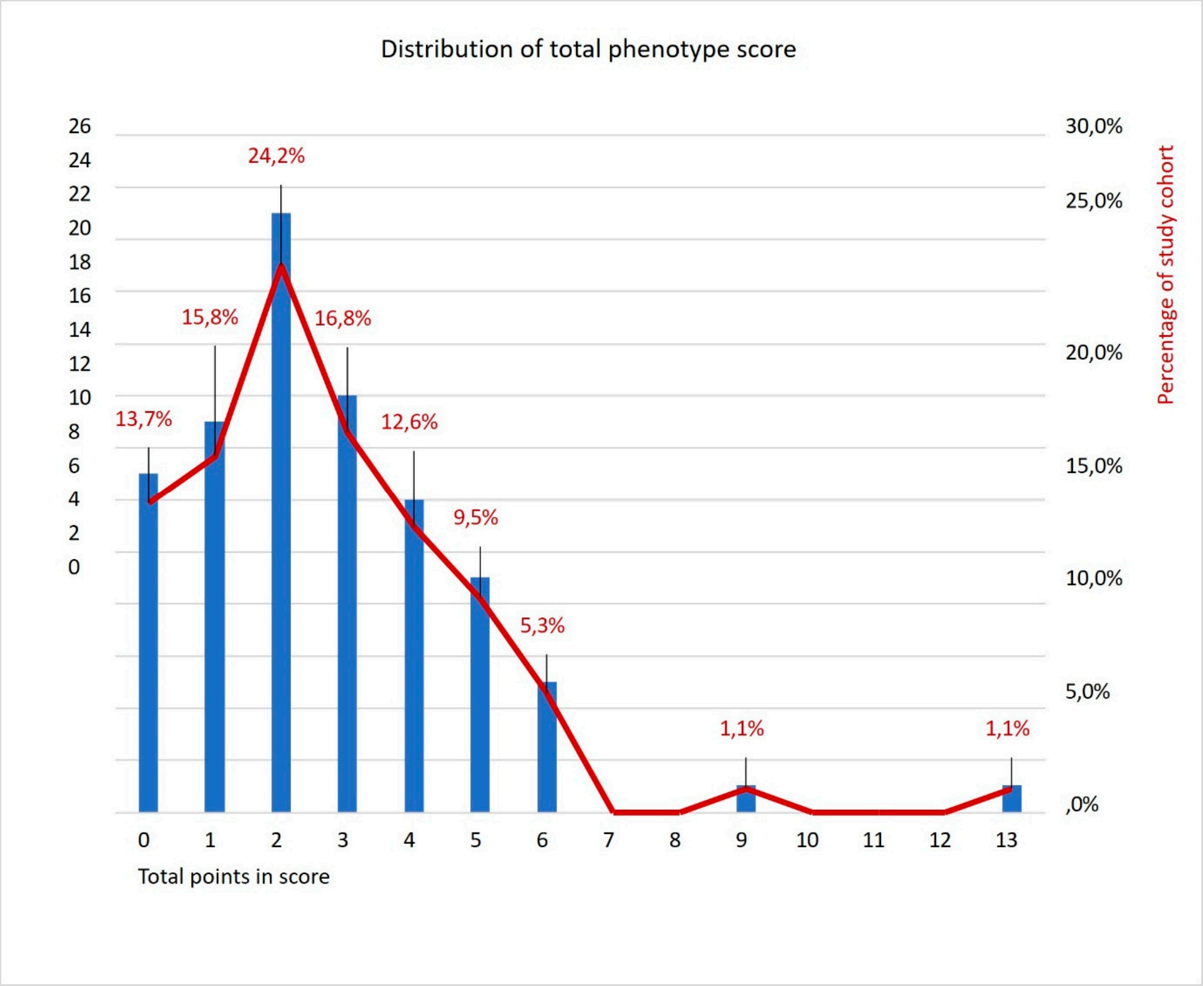

Distribution of total phenotype score
26
24
22
20
18
16
14
12
10
8
6
4
2
0
30,0%
25,0%
20,0%
15,0%
10,0%
5,0%
,0%
Percentage of study cohort
24,2%
15,8%
16,8%
13,7%
12,6%
9,5%
5,3%
1,1%
1,1%
0
1
2
3
4
5
6
7
8
9
10
11
12
13
Total points in score
